# Near-random connections support top-down feature-based attentional modulations in early sensory cortex

**DOI:** 10.1371/journal.pcbi.1013396

**Published:** 2025-08-12

**Authors:** Sunyoung Park, John T. Serences

**Affiliations:** 1 Department of Psychology, University of California, San Diego, La Jolla, California, United States of America; 2 Neurosciences Graduate Program, University of California, San Diego, La Jolla, California, United States of America; UT Austin: The University of Texas at Austin, UNITED STATES OF AMERICA

## Abstract

Top-down feedback from prefrontal cortex (PFC) can enhance the gain of feature selective neurons in early sensory areas that are tuned to behaviorally relevant stimuli (termed *feature-based attention*). Importantly, feature-based attention can even modulate the gain of neurons that do not respond directly to the spatial location of the relevant stimulus, a phenomenon that is thought to globally prime sensitivity to detect relevant features – irrespective of their location – during visual search. However, the neurons in PFC that are thought to provide top-down feedback typically have high-dimensional tuning for multiple features, so it is unclear how feedback selectively modulates responses in neurons tuned to a relevant stimulus without incidentally causing interference by co-modulating neurons tuned to irrelevant features. To investigate this issue, we adapted a spiking neural network model with a first ‘sensory’ layer composed of neurons selective for single features. Neurons in a second ‘control’ layer formed random and reciprocal connections with different sensory neurons, giving rise to PFC-like high-dimensional feature tuning. Stimulating second layer neurons that responded robustly to a relevant stimulus led to corresponding gain modulations in sensory neurons that were directly driven by a relevant stimulus. Importantly, no spurious stimulus-like representations arose in unstimulated sensory neurons – despite high-dimensional tuning in second layer neurons – because the random connections averaged out feedback targeted on irrelevant features. Next, we show that subtly increasing the probability that similarly tuned sensory neurons converge on the same second layer neurons can yield most of the noise-cancelling benefits of completely random connections while simultaneously producing spatially global feature-selective modulations in unstimulated sensory neurons. Collectively, these results suggest that a delicate balance between randomness and structure can support top-down feedback signals that globally enhance sensory neurons tuned to relevant features, without leading to spurious stimulus representations that might interfere with perceptual processing.

## Author summary

In everyday life, we focus on what matters—like finding our car keys on a messy desk—by sending signals from higher control brain areas to earlier sensory brain areas. These “top-down” signals help us better process important information while ignoring distractions. While there is some structure in the connections given the precision of the signals, many neurons involved in sending these top-down signals respond to a wide range of features, not just the relevant ones. Would this broad – and seemingly random – tuning cause the brain to accidentally enhance irrelevant information as well? Using a spiking neural network model we found that even when feedback signals came from neurons tuned to many different features, they still sharpened responses to the relevant input without producing misleading signals about irrelevant inputs. However, this cancelling of irrelevant signals is only observed when connections between control and sensory areas are close to random, as near-random overlap between connections averages out most unwanted signals. We conclude that a balance between structured and random connections may allow the brain to deliver precise feedback even with broadly tuned neurons, helping to process only the most relevant sensory inputs.

## Introduction

Efficient visual search requires selectively prioritizing stimulus inputs based on top-down knowledge of currently relevant features [1–3]. Although it is well-established that behavioral goals can modulate the gain and covariance structure of neural codes in early sensory cortex [4], fundamental questions remain about how top-down modulations back-propagate from higher-order areas of pre-frontal and parietal cortex to influence perception [5–8]. For instance, basic visual features such as colors or orientations are encoded by neurons in striate and extrastriate visual cortex (EVC) that are tuned to specific feature values and have relatively small spatial receptive fields [9–13]. In contrast, neurons in PFC have much larger spatial receptive fields and encode more abstract information about behavioral goals [14–21]. Moreover, neurons in PFC often exhibit high-dimensional tuning functions that can encode multiple feature values (termed *mixed selectivity*; [22–27]). Nevertheless, despite being tuned to multiple features, both correlational and causal studies suggest that PFC neurons with high-dimensional selectivity can generate precise modulations that target specific sub-populations of neurons in EVC to enhance the response evoked by just the relevant sensory feature [28–34]. Here we adapted a spiking neural network [35] model to investigate how PFC neurons with high-dimensional tuning can generate precise top-down modulatory signals to facilitate efficient information processing in early visual cortex.

Classic studies in visual information processing demonstrate that EVC neurons are highly selective for specific visual features. For example, invasive recordings reveal that neurons in the striate cortex and EVC typically have small spatial receptive fields, have retinotopic organization, and exhibit selectivity for specific orientations, colors, motion directions or other low-level features [9–13,36–44]. Thus, visual features in different locations are encoded by different subsets of feature-selective neurons in EVC, and with progression up the processing hierarchy, neurons respond to increasingly complex conjunctions of features and spatial receptive fields grow in size [45–53].

While the afferent flow of information through the visual system is relatively well understood, feedback signals – particularly highly precise signals – is not. This is a difficult problem because relaying top-down signals from neurons with complex feature selectivity and large spatial receptive fields would intuitively yield imprecise modulations in early sensory neurons with highly selective tuning. Nevertheless, when a visual feature is behaviorally relevant, top-down attention can selectively enhance the overall speed and accuracy of processing relevant features [54–58], and this processing advantage is thought to be supported by the seemingly precise feedback signals that modulate activity in EVC neurons that have spatial or featural tuning matching the relevant feature [2,4,28,32,33,59–75]. For example, searching for a specific feature, such as upward motion, will increase the firing rate of neurons in MT that are selective for the relevant direction while simultaneously inhibiting the firing rate of neurons tuned to other directions [28,33,76,77]. Importantly, feature-based attention also modulates neurons that have spatial receptive fields far from the location of the relevant stimulus and are thus not directly stimulated. This *spatially global* effect of feature-based attention is typically smaller than the effect on neurons that are directly driven by the stimulus [28]. However, spatially global modulations are thought to be important for priming sensitivity to detect potentially relevant stimuli no matter where they appear in the visual field [3,77–79].

These highly specific top-down modulations of EVC responses are thought to be generated in regions of frontal and parietal cortex, such as the lateral intraparietal area (LIP), the frontal eye fields (FEF), and lateral prefrontal cortex (LPFC; [1,2,6,7,20,80–88]). For example, microstimulation of neurons in the FEF leads to a spatially selective increase in the firing rate of V4 neurons that encode relevant features, mimicking the effects of spatial attention [31]. In addition, while FEF neurons are not inherently selective for stimulus features such as color or orientation [21,89,90], they have been found to flexibly encode different features on the basis of their current behavioral relevance [90–93]. More recently, the ventral prearcuate (VPA) region of the PFC in nonhuman primates has been suggested to compute feature-selective signals that are relayed to FEF to guide the selection of relevant features, as optogenetic inactivation of the VPA reduces feature-based selection effects in FEF and V4 [94,95]. Finally, neurons in LPFC are thought to integrate sensory input and internal states (task rules, behavioral goals) to flexibly control selective attention as a function of current behavioral demands [14,20,96–98]. Analogous observations have been made in human subjects: Damage to LPFC leads to deficits in task switching and the use of complex rules to guide behavior [99–106], disrupting LPFC with transcranial magnetic stimulation impairs top-down control over stimulus-driven responses [107,108], and human imaging studies implicate several prefrontal areas such as the inferior frontal junction (IFJ) in feature-based attentional control (with IFJ potentially homologous with VPA in nonhuman primates) [109–117].

Importantly, the PFC neurons that are thought to play a role in attentional control also typically exhibit mixed selectivity for multiple features that linearly or nonlinearly combine to determine the final output of the neuron. Mixed selectivity can flexibly support representations of different external stimuli and task demands, and can increase the encoding capacity of population responses [23,26,27]. However, these complex responses stand in contrast to the highly selective tuning properties observed in EVC neurons, posing a challenge for understanding top-down attentional control for visual features: How can neurons with high-dimensional tuning in PFC evoke highly selective attentional modulations for a single relevant feature in EVC? For example, feedback signals emanating from a PFC neuron that is sensitive to red and vertical may cause spurious modulations of task irrelevant features in situations where only red is behaviorally relevant. Such unintended modulations may interfere with the specificity of top-down modulations of the target feature or even cause perceptual interference in the form of illusory percepts.

**Fig 1:**
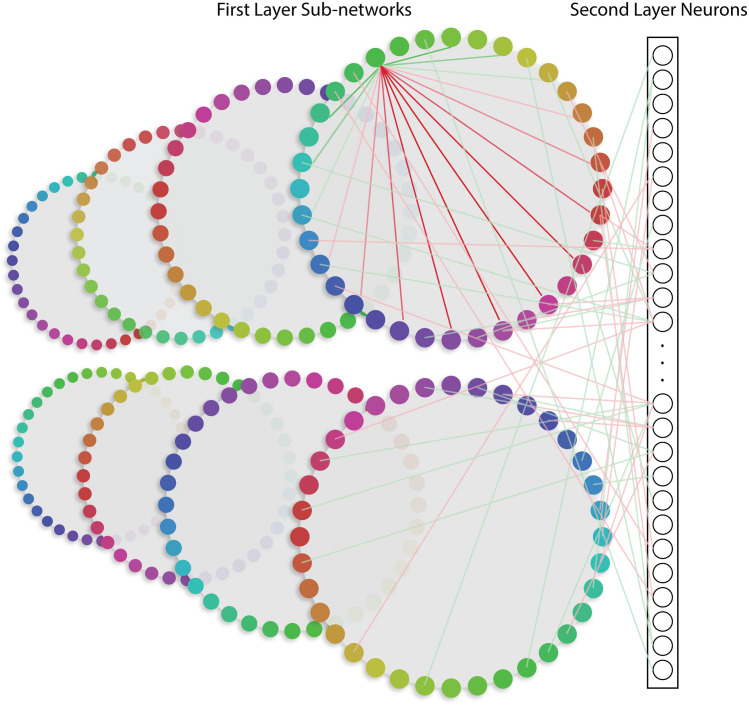
Overview of spiking network model adapted from Bouchacourt & Buschman (2019) [35]. The model had two layers of Poisson spiking neurons. The first layer contained 8 ring-like ‘sensory’ sub-networks that each contained 512 neurons, simulating sensory networks that encode information in circular stimulus spaces. Every neuron in a sub-network had a preferred stimulus input, with the tuning determined by short-range excitatory connections (green lines within the top-right sub-network) and long-range inhibitory connections (red lines within the top-right sub-network). Each sub-network can be thought of as encoding a specific feature class (e.g., different colors), with each of the 8 sub-networks encoding information from a different spatial position in the visual field. The second layer was composed of 1024 neurons that were randomly and reciprocally connected to the neurons in the first layer (red and green lines connecting to the bottom-right sub-network), with the connections balanced between excitatory (green lines) and inhibitory (red lines). Importantly, a second layer neuron could be connected to sensory neurons in multiple sensory sub-networks (but for clarity, only connections between the first two sensory sub-networks and the second layer are shown here). Thus, even though the first layer sub-networks were not directly connected to each other, they could indirectly interact via feed-forward and feedback connections to the second layer. Stimulus inputs were a circular normal distribution centered on an angle between 1° and 360°. Figure adapted from [35].

One solution for this correspondence problem involves collapsing the high-dimensional tuning functions of PFC neurons before relaying modulatory signals to EVC. However, this solution is challenging as the neurons generating the control signals would need an additional control signal directing how to collapse their selectivity. Here, we investigate an alternative hypothesis that a high degree of randomness in the feedforward and feedback connections between EVC and higher-order regions largely cancels out spurious signals while preserving the desired modulatory signals targeting the relevant feature. To test this hypothesis, we adapted a spiking neural network model [35] to simulate an architecture in which several sub-networks of ‘sensory’ neurons with unimodal selectivity for a single stimulus feature interact with higher-order ‘control’ neurons that exhibit high-dimensional selectivity for multiple stimulus features (Fig 1). While stimulating the control neurons in the model reproduces commonly observed feature-based attention effects in sensory neurons, top-down attention also causes systematic modulations in unstimulated sensory neurons due to the high-dimensional tuning of the control neurons. We then demonstrate that this unintentional spread of top-down modulations does not lead to spurious stimulus-like responses due to the unstructured nature of the connections between sensory and control neurons. This observation suggests that fully random inter-layer connections may effectively cancel out spurious stimulus-like responses that would otherwise impair perception. Finally, we show that adding a modest amount of structure to the between-layer connections mostly preserves the benefits of fully random connections while also recapitulating other empirically observed phenomena like the spatially global spread of feature-based attention [28,33,118,119].

## Results

We adapted a spiking neural network architecture that was previously used to model storage capacity and patterns of errors in visual working memory [35]. We adapted this model because it has highly tuned sensory neurons that provide converging inputs to higher dimensional second layer neurons, and because it can maintain a stable representation of a relevant stimulus even in the presence of an irrelevant competing stimulus (with some minor modifications, see Methods). The model consisted of two layers of Poisson spiking neurons. The first ‘sensory’ layer consisted of 8 sub-networks, where each neuron in a sub-network had short-range excitatory connections and long-range inhibitory connections to other neurons within the same sub-network. As a result, all sensory neurons had circular normal tuning functions centered on a single feature value. In the second ‘control’ layer, each neuron was randomly connected via reciprocal feedforward and feedback connections to neurons in different first layer sub-networks, with overall excitatory and inhibitory connections balanced to prevent runaway excitation in the network. While the reciprocal nature of the connections is a simplifying assumption, evidence suggests that such long-range connections occur more often than chance, particularly at the level of regions (reviewed in [120]). This architecture gives rise to two important properties: (1) neurons in the second layer had high dimensional tuning for multiple features via converging inputs from first layer neurons with different tuning preferences, and (2) even though the first layer sub-networks were not directly connected to each other, neurons in different sub-networks indirectly interacted via overlapping feedforward and feedback connections with neurons in the second layer. This latter point is key to testing our central hypothesis about the role of random between-layer connections in supporting highly precise attentional modulations when top-down feedback is relayed via second layer neurons with high-dimensional selectivity. Note that second layer neurons in this network exhibit linear mixed selectivity due to converging sensory inputs, not nonlinear selectivity that can vary as a function of task demands or context. However, linear mixed selectivity is sufficient given our focus on understanding how top-down signals can be effectively relayed via control neurons tuned to many features (see also Discussion). Moreover, although the second layer was designed to express higher-dimensional selectivity due to converging sensory inputs, it is not intended to represent a specific brain area, such as VPA or LPFC, but rather any region where cells with complex tuning properties provide top-down feedback to early sensory cortices.

### Basic feature-based attention effects in the network

To establish a method for inducing top-down feature-based attention effects in the network, we modeled our task after prototypical feature-based attention experiments in which two spatially overlapping stimuli are presented while attention is switched between the two stimuli based on task instructions [75,79,119]. For example, Serences & Boynton (2007) [119] presented two spatially overlapping moving dot fields to the left of fixation, with one dot field moving at 45° and the other at 135°. Depending on a centrally presented cue, subjects had to attend to either the 45° or the 135° dot field to detect a relevant target. Taking measurements from visual areas that had spatial receptive fields overlapping the moving dots revealed a selective enhancement of responses to the attended compared to the unattended stimulus. Moreover, measurements from visual areas that responded to unstimulated regions of the visual field also revealed modest feature-selective modulations, suggesting a spatially global spread of feature-based attention (see also: [28,33,77,79,118]).

**Fig 2:**
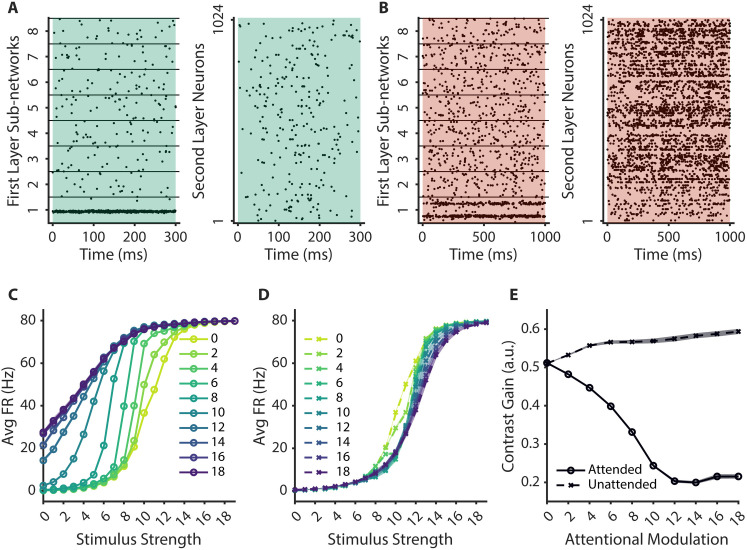
Feature-based attention simulations. **A.** An example trial from the *sensory* task in which sub-network 1 was presented with a stimulus input at 157.5° for 300ms. There were 16 possible stimulus values presented in this sensory task, with the stimulus values uniformly tiling the full stimulus space. **B.** An example trial from the *attention* task. Two stimulus inputs, at 90° and 270°, were presented to sub-network 1 and at the same time (stimulus strength: 6), feature-based attentional modulation was applied to a subset of second layer neurons that have the highest selectivity to the 90° stimulus in sub-network 1 (lower cluster of spikes; attentional modulation strength: 8) for 1000ms. **C, D.** Response functions for when the stimulus was attended (**C**) and unattended (D). Each line represents a different level of feature-based attentional modulation strength. **E.** Estimated contrast gain parameters from Attended (solid black line) and Unattended (dotted gray line) conditions. Shaded areas in **C, D, E** represent standard error of mean across 10 different network initializations.

Before fully implementing a version of this attention task that involves competition between two spatially overlapping stimuli, we first used a *sensory* task in which a single stimulus was presented to the first sensory sub-network to drive feature-specific activity patterns using 16 different stimulus values to uniformly tile the span of the stimulus space (Fig 2A). Based on the averaged evoked response during the last 250ms of each trial, we identified 20% of the second layer neurons that responded maximally to each stimulus. Then we implemented a version of the *attention* task where we simultaneously presented two stimuli (90° and 270°) to the first sensory sub-network, with top-down feature-based attention simulated by applying varying levels of modulation to the 20% of second layer neurons that were identified in the sensory task as being maximally responsive to one of the two stimuli in the attention task (Fig 2B, see also S4 Fig). While this method of determining which neurons in the second layer to target with top-down modulation required some knowledge about network connectivity, it was inspired by demonstrations that global neuromodulators like norepinephrine interact with local ‘hot-spots’ of stimulus-driven activity to amplify neural gain and feedback signals to early sensory areas (termed the GANE model, see Discussion and [121]).

To determine if our manipulation of feature-based attention modulated firing rates in the first sensory sub-network, we first quantified the average responses of neurons that were tuned to the attended and unattended stimulus as a function of stimulus strength and the strength of the top-down attentional modulation (Fig 2C, 2D). We observed a gain modulation such that response functions were systematically shifted leftward with increasing top-down attentional modulation, indicating heightened sensitivity to lower-strength stimuli (Fig 2C) [54,72,122]. In contrast, response functions associated with unattended stimuli shifted rightward when stronger top-down modulation was applied to the attended stimulus due to the inhibitory connections between neurons tuned to the attended and unattended stimuli in the stimulated sensory sub-network (Fig 2D). We then quantified these shifts by fitting the response functions with a Naka-Rushton equation, where the contrast gain parameter of the model is inversely related to changes in sensitivity (i.e., leftward shifts in the response functions, see Methods). As shown in Fig 2E, we observed a diverging pattern of contrast gain parameters such that higher attentional modulation increased sensitivity for the attended stimulus and slightly decreased sensitivity to the unattended stimulus (indicated by decreasing and increasing contrast gain parameters, respectively). These results demonstrate that our network captures basic feature-based attention effects by showing an increase in neural activity evoked by relevant features compared to irrelevant features.

### Assessing spurious stimulus-like representations in unstimulated sensory sub-networks

Given that the second layer neurons could have connections with sensory neurons in multiple sub-networks, we next assessed whether top-down attentional signals might induce spurious stimulus-like representations in unstimulated sensory sub-networks. To assess if any such modulations resembled the activity patterns evoked by real sensory stimuli, we used the single-stimulus *sensory task* described in the last section to train decoding models on the stimulus-driven activity patterns during the last 250ms of each trial in the stimulated and in each of the unstimulated sensory sub-networks. Due to the continuous nature of our stimulus space, we selected circular ridge regression as our decoding model. The decoding models for each sub-network were then applied to response patterns observed during the attention task to predict which of the two stimuli was attended in the first sensory sub-network (using a fixed stimulus strength of 10 for the attention task to match the stimulus strength used in the sensory training task, see S1 Fig for all stimulus strengths). This approach allowed us to determine if the attentional gain modulations reported in the previous section enhanced the amount of stimulus-specific information encoded in the stimulated sub-network. More importantly, we could assess whether similar stimulus-specific representations incidentally arose in unstimulated sub-networks due to second layer neurons simultaneously sending feedback signals to many sensory sub-networks.

**Fig 3:**
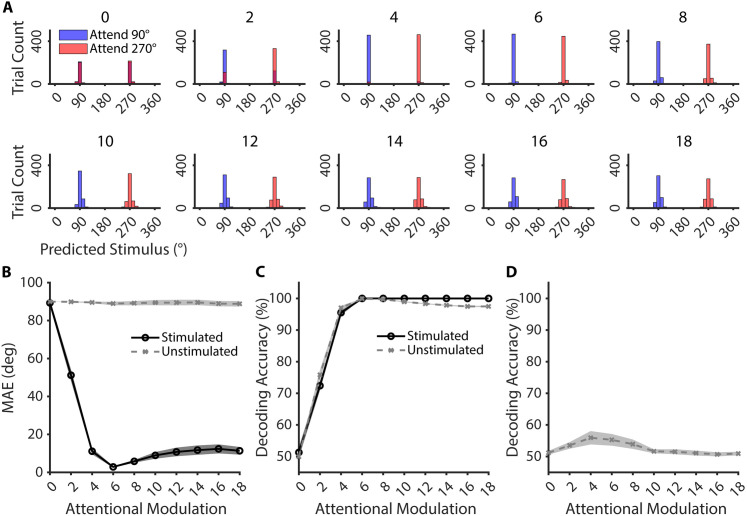
Decoding results from feature-based attention simulations for trials where stimulus strength was fixed at 10. **A.** Histograms of the predicted attended stimulus values for every trial in the *attention* task for the stimulated sub-network based on a circular ridge regression model trained on the *sensory* task, collapsed across all network initializations. Trials in which feature-based attention was directed to the 90° stimulus are shaded in purple (Attend 90°), and the trials in which attention was directed to the 270° stimulus are shaded in red (Attend 270°). Each histogram represents predictions from different levels of feature-based attentional modulation strength as indicated above each plot. **B.** Average MAE between the predicted and actual stimulus inputs for stimulated (solid black line) and unstimulated (dotted gray line) sub-networks based on decoding model predictions. **C.** Decoding accuracy of support vector machines (SVMs) trained and tested on the *attention* task for Stimulated (solid black line) and Unstimulated (dotted gray line) sub-networks. **D.** Decoding accuracy of SVMs based on the *attention* task, trained on one unstimulated sub-network and tested on another unstimulated sub-network. Shaded areas in B, C, D represent standard error of mean across 10 different network initializations.

We plotted the frequency of classifier predictions for each trial as histograms, with trials in which the attentional target was 90° in purple and the trials in which the attentional target was 270° in red (Fig 3A). Using activity patterns associated with the stimulated first sensory sub-network, the decoding model was equally likely to predict that either of the two presented stimuli was attended in the absence of top-down attention (Fig 3A, top-left panel). The decoding model was increasingly accurate at predicting the value of the attended stimulus as we increased the strength of attentional modulation to a moderate level (Fig 3A, attention strengths of 2–6). However, predictions grew increasingly more dispersed around the attended target at even higher levels of top-down attentional modulation (Fig 3A, attention strengths of 8 and higher). This increase in prediction variability was caused by overly strong top-down attentional modulations saturating the firing rates of neurons that encode the attended feature and spilling over to neighboring neurons, rendering the response patterns more diffuse and increasing decoder error. This change in prediction accuracy is reflected in systematically decreasing mean absolute prediction errors (MAEs, Fig 3B, solid black line) as a function of attentional modulation strength.

The results in Fig 3A establish that top-down attention generally enhances the precision of neural activity patterns associated with attended stimuli in the stimulated sub-network. Next, we examined decoding precision in unstimulated networks to see if coherent, stimulus-like, representations were induced by the spillover of top-down attention relayed by second-layer neurons that were connected to multiple neurons in multiple sensory sub-networks. As shown in Fig 3B, decoding models trained on sensory-evoked patterns in each of the unstimulated sub-networks were at chance, irrespective of top-down modulation strength, demonstrating that stimulus-like modulations are contained to the stimulated sub-network. More importantly, the chance decoding in the unstimulated networks demonstrates that randomness in feedforward and feedback connections largely cancels out spurious, stimulus-like, representations of irrelevant stimuli that might directly lead to perceptual interference.

### Assessing idiosyncratic representations in unstimulated sensory sub-networks

Even though top-down attentional modulations did not give rise to coherent, stimulus-like, representations of irrelevant stimuli in the unstimulated sub-networks, we next tested to determine if there were idiosyncratic, yet consistent, feedback modulations relayed to the unstimulated sub-networks that might give rise to reliably decodable activity patterns. Because we posited that the feedback patterns would not resemble stimulus-evoked responses due to the random nature of the connections, we opted to use a support vector machine (SVM) decoder as opposed to the circular regression decoder used in the previous section (as circular regression models assume a continuous feature space). We trained binary SVMs to classify whether the attended stimulus was 90° or 270° based on (1) activity patterns in the stimulated sub-network, and (2) activity patterns in each of the unstimulated sub-networks (with separate SVMs trained/tested for each sub-network using cross-validation). Decoding accuracy based on activity patterns in the stimulated sub-network increased with stronger top-down attentional modulation (Fig 3C, solid black line), consistent with the regression-based MAE results shown in Fig 3B. Critically, activity patterns in the unstimulated sub-networks also supported decoding of the attended stimulus feature, with the average decoding accuracy across all unstimulated sub-networks increasing as a function of increasing top-down attentional modulation (Fig 3C, dotted gray line).

Since the connections between the two layers were random, the spread of top-down modulations to the unstimulated sensory sub-networks should also exhibit idiosyncratic patterns that are different across sub-networks. To confirm that changes in activity patterns in the unstimulated sub-networks were indeed driven primarily by random patterns of feedback, we next compared attention-dependent activity patterns across different unstimulated sub-networks. We trained a SVM on the activity patterns from one unstimulated sub-network and cross-generalized that classifier to the other unstimulated sub-networks, repeating over all possible training and testing pairs of unstimulated sub-networks. We predicted that decoding accuracy should remain close to chance regardless of the strength of the top-down modulations since each sub-network should have random, and thus independent, connections with the second layer. Alternately, above-chance cross-generalization across different unstimulated sub-networks would suggest a shared representation that covaries with the attended stimulus in the stimulated sub-network. Consistent with independent attentional modulations, the average cross-sub-network decoding accuracy was close to chance, even at the highest levels of top-down attentional modulation (Fig 3D).

Together, the results in Fig 3C, 3D suggest that relaying top-down modulations through high-dimensional neurons leads to systematic modulations in unstimulated networks. However, the randomness of the feedback connections ensures that incidental feedback signals largely cancel out due to destructive interference. This destructive interference allows only the modulatory signals targeted on the relevant feature in the stimulated sub-network to pass, while preventing the unintended emergence of stimulus-like representations in unstimulated sub-networks that might interfere with efficient information processing by causing spurious, stimulus-like representations.

### Introducing partially structured connections can account for global feature-based attentional modulations

The random connections between the first and second layers of the network supports highly precise top-down modulations and consistent, but not stimulus-like, modulations in the unstimulated networks. Thus, random connections are potentially useful as that they prevent the emergence of representations of irrelevant stimuli that might interfere with perception. However, prior empirical studies have demonstrated global feature-based attention effects such that the response of a neuron is modulated based on the similarity between its preferred feature and the attended feature, irrespective of the location of the neuron’s spatial receptive field [28,33,78,79,118]. These modulations are smaller in magnitude than responses evoked by physically present sensory stimuli but nevertheless are thought to play an important role in increasing sensitivity to relevant features across the entire visual field [28,77–79]. To determine if our model could produce feature-based modulations of the relevant behavioral feature in unstimulated sub-networks, we systematically decreased the randomness of the between layer connections to determine if intermediate levels of structure could simultaneously preserve desired modulations in the stimulated network while still giving rise to a global feature-based attention.

**Fig 4:**
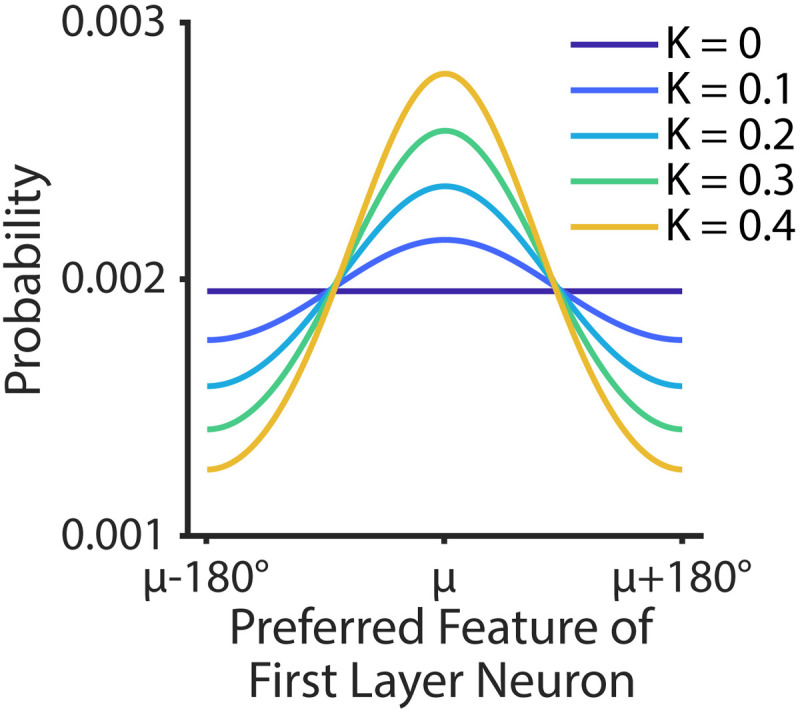
Probability distribution of connections between a second layer neuron and first layer neurons in different sub-networks as a function of the preferred feature of the first layer neurons. For every second layer neuron, the center of the probability distribution (μ) was randomly chosen from all possible values in the stimulus space. Each line represents a circular normal distribution with different levels of the concentration parameter κ, as shown in the legend.

In a fully random network as described in the preceding sections, no constraints were placed on the formation of connections between the two layers (except those governing the overall probability of forming any connection at all, see Methods). In contrast, in a highly structured implementation of the network, if a second layer neuron had a connection with a neuron from one sensory sub-network that was tuned to 180°, that second layer neuron would also be more likely to have a connection with a neuron in another sensory sub-network that was also tuned to 180°. If we interpret each sub-network as encoding information from a different spatial location, then this structure would lead to the second layer neuron developing a preference for 180° stimuli across the visual field. To parametrically manipulate the degree of structure in the connections, we adjusted the concentration parameter (κ) of a circular normal distribution that determined the probability that a given second layer neuron was connected to similarly tuned first layer neurons across different sensory sub-networks (Fig 4). For example, when the connections were fully random (κ = 0), all sensory neurons from all sub-networks had an equal probability of being connected to a given second layer neuron. In contrast, when connections were more structured (κ = 0.4), the network became less random because there was a higher probability of connections between similarly-tuned sensory neurons converging on a given second layer neuron.

We predicted that decreasing the randomness of cross-layer connections (by increasing the concentration parameter) would change the network behavior in two ways: (1) second layer neurons would have lower dimensional tuning because the sensory neurons that provide feedforward input would have similar feature tuning preferences, (2) stimulus inputs to one sub-network would modulate activity in unstimulated sub-networks in a feature-selective manner, in contrast to the consistent, but idiosyncratic, modulations observed in a fully random network.

**Fig 5:**
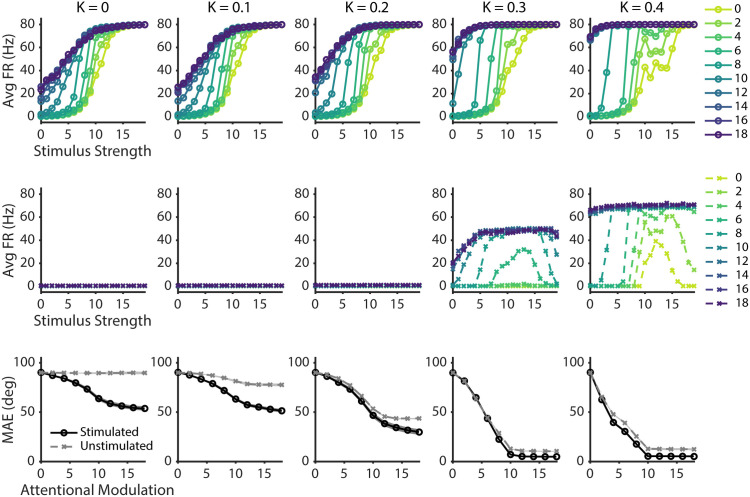
Manipulating randomness in between-layer connections. Each column represents results from different level of connectivity randomness (κ), as marked at the top. Top row shows response functions for the attended stimulus in the stimulated sub-network. Middle row shows response functions in the unstimulated sub-network, based on firing rates of the neurons that prefer the attended feature. The color of each line represents different levels of feature-based attentional modulation strength, as shown in the legend. The bottom row shows average mean absolute error (MAE) between the predicted and actual stimulus input for stimulated (solid black line) and unstimulated (dotted gray line) conditions based on regression model predictions. Shaded areas represent standard error of the mean across 10 different network initializations.

As a first step to assess the emergence of stimulus-specific representations in unstimulated sub-networks as a function of network structure (κ), we measured the average firing rates of neurons that maximally prefer the attended stimulus in the stimulated sub-network (Fig 5, top row) and in each of the unstimulated sub-networks (Fig 5, middle row). We found that responses evoked by the attended stimulus in the stimulated sub-network generally shifted leftward when there was more structure in the between-layer connections (i.e., higher concentration parameter κ). This shift likely occurred because similarly-tuned neurons in different sensory sub-networks were more likely to indirectly interact via common connections with the same second layer neurons, recursively amplifying the effects of attentional modulations. Importantly, even though there was no external stimulus input provided to the unstimulated sub-networks, neurons that were tuned to the attended feature also exhibited an increase in structured activity centered on the attended feature (i.e., at κ = 0.3 and 0.4). This effect was most pronounced for higher feature-based attentional modulation strengths and attentional modulation strengths of 8 or higher. Interestingly, when κ = 0.4 and at intermediate stimulus strengths, we observed increased activity in the unstimulated sub-networks even when no top-down attentional modulation was applied (attentional modulation = 0, light green line). Thus, a high degree of structure in the connections between like-tuned neurons in the first layer sub-networks and second layer neurons allowed strong stimulus-driven activity in one sub-network to indirectly evoke similar activity patterns in other sub-networks. Moreover, at high values of κ we found a drop in the magnitude of responses evoked by the attended stimulus when stimulus strength was high and top-down attentional modulation strength was low. This drop was likely due to top-down attentional modulations not being robust enough to overcome strong competing signals from the unattended stimulus.

To further quantify whether representations in the unstimulated sub-networks resembled stimulus-driven response patterns, we performed a cross-generalization decoding analysis by training a circular ridge regression model on the single-stimulus *sensory* task to predict the attended stimulus in the *attention* task, as described in preceding sections. When the between-layer connections were slightly more structured (i.e., κ = 0.1), MAE for the stimulated sub-network showed a similar pattern to when the connections were fully random, with decreasing MAE as a function of increasing top-down attentional modulation (Fig 5, bottom row). Importantly, in the unstimulated sub-networks, there was a slight decrease in MAE with stronger top-down attentional modulation, although it was smaller than in the stimulated sub-network. This suggests that when the network has a low level of structured connections between layers, feedback signals can modulate activity in unstimulated sub-networks in a way that resembles bottom-up stimulus-driven representations. However, the stimulus-like representations in the unstimulated sub-networks were not as coherent as the representations in the stimulated sub-network, mirroring empirically reported global feature-based attention effects [28,33]. Thus, with low levels of structure in the connections, the weak stimulus-like representations in unstimulated networks may still be strong enough to prime the detection of relevant features in unattended spatial locations without being overly strong and causing interference (or illusory percepts).

As we further increased the structure of the connections, with κ = 0.2 and higher, MAE in the stimulated sub-network decreased even more with stronger top-down attentional modulation. This pattern suggests that the activity in the stimulated sub-network increasingly resembled the activity pattern of when the attended stimulus was presented without a competing irrelevant stimulus. However, MAE in the unstimulated sub-networks also dropped precipitously to levels similar to the MAE in the stimulated sub-network. This pattern implies that the feedback-modulated activity patterns in the unstimulated sub-networks closely resembled the stimulus-driven representations due to the indirect spread of activity between first layer sub-networks. In turn, equally strong stimulus-like representations in both the stimulated and unstimulated sub-networks could lead to interference because a stimulus-driven percept could indirectly evoke illusory percepts via the propagation of signals through second-layer neurons.

## Discussion

While it is widely believed that top-down modulatory signals originate from the prefrontal and parietal cortex, neurons in these areas typically have high-dimensional tuning. As a result, the ability of these neurons to send highly specific feedback signals to neurons in early sensory cortex is not well understood. Here, we used a spiking neural network to test the hypothesis that a balance between random and structured connections between sensory and high-order areas supports targeted top-down feedback without leading to spurious representations that could interfere with perceptual processing. The model produced contrast gain effects with top-down attention, replicating prior empirical findings [2,123]. Importantly, increasing the strength of feature-based attentional signals did lead to idiosyncratic – but highly consistent – modulations in unstimulated sub-networks that did not resemble coherent sensory responses. Thus, neurons with high-dimensional tuning can spread top-down signals throughout hierarchical networks. However, if connections are sufficiently random, then these non-specific signals largely cancel out and do not give rise to coherent representations of irrelevant stimuli that could be mistaken as a real stimulus input. Finally, introducing a modest amount of structure into the inter-layer connections, in the form of a non-uniform probability that like-tuned sensory neurons connect with the same second-layer neurons, can recapitulate empirically observed spatially global feature-based attention effects without leading to overly strong representations in unstimulated sub-networks. Thus, a careful balance between random and structured connections in a hierarchical network supports selective feature-based attention effects while guarding against interference induced by “illusory” stimulus representations.

As pointed out in previous empirical and theoretical work, random connections between early and later processing stages may be important for flexibly encoding information in working memory [124] and decision making during complex cognitive tasks [125,126]. Even though early sensory areas are thought to be more structured and lower-dimensional in their selectivity, random connections can give rise to convergent inputs that results in high-dimensional selectivity in the later areas, supporting the ability to integrate and represent many different types of information [35]. Building on these findings, our results demonstrate the importance of random connections in the context of top-down attentional modulations passed backwards through a hierarchical network. Given the high degree of convergence from early sensory areas to high-order control areas, feedback signals propagated from the control neurons will inevitably spread to many early sensory neurons [20,127]. However, the present results suggest that randomness in these connections plays a key role in selectively enhancing relevant information while canceling out idiosyncratic signals (see also S1 Fig from reference [35]). As noted in references [35,124], one possible mechanism to implement random connections is cell adhesion molecules such as protocadherin that mediate self-avoidance in dendritic connections [128–131]. These proteins are expressed in a probabilistic way in different combinations that give individual neurons random, unique molecular identities. Thus, while speculative, matching the neurons in different brain areas with the same molecular signature may support the initialization of random and reciprocal connections [35,124].

Based on previous studies and our simulations, maximal randomness in a network supports targeted top-down enhancement of relevant sensory responses. However, a fully random network does not naturally express the spatially global spread of feature-based attention, in which a neuron’s activity is amplified based on the similarity between its preferred feature and the attended feature, irrespective of the location of its spatial receptive field [28,33,77–79,118,132,133]. As shown in Fig 5, parametrically changing the structuredness/randomness of between-layer connections revealed a balance point where the unstimulated sub-networks exhibited activity patterns that were similar to patterns in the stimulated sub-network, but at a lower level than in the stimulated sub-network. This emulates the spread of feature-based attention signals to locations where no stimulus was presented [119,132] and may increase sensitivity for the relevant feature across the entire visual field without leading to illusory percepts that out-compete the actual stimulus [119]. As the magnitude of structured connections increases (i.e., higher *k* values in Fig 4), the global feature-based attention effects grew in magnitude to match the magnitude of responses evoked by the stimulus. Like spurious representations of completely irrelevant features, multiple simultaneous representations of the relevant feature in different spatial locations – all with same level of intensity – would also impair perception. Moreover, an overly structured connection scheme would produce low dimensional tuning in higher-order areas that resembles the tuning of early sensory neurons, contrary to empirical observations that many PFC neurons show high-dimensional selectivity to diverse sets of features and task demands [14,23,126,134,135]. Thus, our results suggest that some structure in the connections between early sensory areas and high-order areas may support beneficial modulations like modest global feature-based attention effects. However, too much structure may be deleterious to efficient perceptual processing.

Aside from structured connections between sensory areas and higher-order areas, long-range lateral pathways that transmit excitatory signals between sensory neurons tuned to similar features may also contribute to global feature-based attention. However, while these lateral connections may complement top-down feedback signals, they are likely insufficient on their own to account for the range of empirical observations. Horizontal excitatory connections between similarly tuned neurons can amplify feature-selective signals, but their spatial extent is limited (~6 mm in cat and macaque V1 [136,137]) falling short of covering the entire visual field. In addition, cross-hemispheric connections through the corpus callosum may facilitate interhemispheric coordination [138–140], particularly near the vertical meridian. However, these callosal projections are primarily restricted to midline regions and are not well suited for generating spatially uniform modulations across the visual field. Collectively, existing data thus suggest that structured top-down projections are likely a necessary mechanism involved in producing global feature-based attention effects.

In the current study, we focused on top-down feature-based attention by manipulating a single layer of ‘control’ neurons that was intended to represent any putative control region where neurons exhibit mixed selectivity. This simplified model was sufficient to test our hypotheses about the importance of randomness when relaying feedback signals via neurons with high-dimensional tuning. However, to also account for spatial attention, future work might explore networks with two “second” layers - one with structured connections for spatial attention and one with less structured connections for feature-based attention. For example, FEF neurons have spatial tuning and have been implicated in space-based attentional control [31,141–144]. In contrast, the inferior frontal junction (IFJ) has been implicated in feature/object-based control in humans [109,110,114–117]. In addition, a recent study showed that inactivating VPA in monkeys impaired the ability to saccade to a target in a dense search array while the ability to saccade to a single target was intact, suggesting VPA’s involvement in guiding feature-based attention (and may be similar to IFJ in humans, [94,95]). In turn, signals in VPA could be relayed to FEF to modulate prioritization and orienting to the selected feature in the spatial domain. While there are certainly many architectures to explore, more complex interactions between spatial and feature-based attention may be better addressed with a more complex network model with functionally separate spatial and feature-based control layers.

While the response properties of the second layer neurons in our simulations were intended to mimic some of the complex response properties of PFC neurons, it is difficult to determine if the same cells that participate in feature-based attentional control also exhibit mixed selectivity or if there are distinct populations of interdigitated cells that express each of these properties to differing degrees. For example, Bichot et al. demonstrated that there is consistent stimulus selectivity in ~35% of units in VPA [94]. However, selectivity was assessed by comparing responses to a small set of 8 natural object images, so it is unclear if these cells demonstrate mixed selectivity. Similarly, more recent work by Mendoza-Halliday et al. showed some selectivity for motion direction in the VPA (termed posterior LPFC in their paper), but again the stimulus space was restricted and no direct assessment of mixed selectivity was performed [96]. That said, other studies have shown mixed selectivity for different stimulus/task components in ventral LPFC [145]. While it is challenging to compare across studies to determine if this mixed selectivity occurs in the same neurons that Bichot and others have implicated in the control of feature-based attention, the recording sites are generally consistent (posterior LPFC, just below the principal sulcus). Thus, it seems likely that cells with mixed selectivity are at least interdigitated with the VPA neurons that are thought to play a role in mediating feature-based attention. That said, additional work is required to better characterize the tuning properties of cells in this general region of ventral LPFC to determine if the same cells that play a role in the control of feature-based attention have complex and flexible tuning properties that are analogous to the second layer neurons used in the present simulations.

Although we focused here on varying the degree of randomness in connections mediating feature-specific feedback, we can speculate about how varying the structure of connections mediating both spatial and featural feedback might support flexible attentional control. For example, even in the more ‘structured’ scenarios in the current model, a given pair of second layer neurons that are both connected to the same sensory sub-networks – giving them similar spatial tuning – could have entirely unrelated tuning for features. This is consistent with some empirical evidence suggesting that LPFC neurons in particular have large spatial receptive fields but highly flexible feature tuning that depends on task demands [23,26]. That said, it seems likely that the degree of stability/flexibility varies both across areas and even within areas, as neurons in LPFC can simultaneously exhibit both ‘classical selectivity’ that reflects stable tuning as well as more flexible selectivity that carries information about relevant stimuli/tasks even when the classical selectivity is removed [26]. In the present context, the diversity of response properties in PFC might provide exactly the type of flexibility required to shift along a continuum from random to structured feedback depending on task demands and tolerance to spurious spatial and featural activations in sensory cortex. For example, targeting second layer neurons for top-down drive solely based on their spatial tuning would lead to spatially specific modulations in sensory neurons. However, differences in feature-tuning in the population of second layer neurons would null out any feature specific modulations. In turn, if second layer neurons were tuned to a common spatial location and to a common feature, then the network would produce highly focal spatial and featural modulations. Thus, having higher-order neurons with flexible tuning for spatial positions and features, either within or between brain regions (e.g., FEF vs LPFC vs VPA), might provide a mechanism to dynamically adjust the degree of randomness in the feedback signals based on the relative utility of having focused vs diffuse space or feature-based attentional modulations.

Our study focused on relaying top-down modulations and did not address the deeper question about how the modulations are initialized. One model that motivated our approach holds that norepinephrine released from the locus coeruleus interacts with stimulus-driven local glutamatergic signaling in areas like parietal cortex and PFC to enhance high priority representations [121,146,147]. In this “glutamate amplifies noradrenergic effects” (GANE) model, high levels of local glutamate associated with the stimulus representation can lead to enhanced release of local norepinephrine to form “hotspots” that are maintained through a positive feedback loop. At the same time, the locus coeruleus inhibits representations that do not form these hotspots via increased lateral inhibition, resulting in a widespread suppression of low-priority representations. This model provides one viable explanation for how top-down modulations might be initialized without the need to hypothesize a hierarchical series of control areas. However, the mechanism that mediates the initial prioritization of a relevant location or feature to start the GANE positive feedback loop that forms hotspots is still unclear.

The higher-order neurons in our network expressed linear mixed selectivity. However, random connections may play a role in canceling out the spreading of spurious activations from higher-order neurons with nonlinear mixed selectivity as well. For example, suppose that a network had two configurations of random connections, one configuration for task A and another for task B. This would result in nonlinear mixed selectivity in the second layer neurons such that tuning for task A would not predict tuning for task B. However, if the connections were largely random in each task configuration, unwanted modulations would still cancel out and only the signal relevant to the target stimulus would be enhanced by top-down attention. Thus, we speculate that random connections play an important role in controlling spurious activations when higher-order neurons with high-dimensional tuning modulate sensory neurons, regardless of whether they exhibit linear or nonlinear mixed selectivity.

With fully random connections, the idiosyncratic patterns we found in unstimulated sub-networks were consistent with respect to the attended feature in the stimulated network. As a result, the response patterns in unstimulated sub-networks supported decoding of the attended feature even though the response pattern did not resemble a coherent, stimulus-evoked response. This may explain some previous findings reporting successful decoding of an attended feature from cortical areas that respond to an unstimulated part of the visual field [119]. While feature-specific modulations could co-occur with the idiosyncratic feedback signals, the consistent, but random, signals may nevertheless contribute to previous observations of global feature-based attention effects. More generally, this observation highlights the fact that significant classifier decoding accuracy only establishes the presence of information but says little about the format of the information. Instead, explicit encoding models should be used whenever possible to better understand the type of code that is being expressed in a neural population [148,149].

While highly variable, empirically reported modulations of mid-level visual areas are typically reported to be on the order of 10–20% [29,150,151]. In our simulations, that corresponds to attentional modulation strengths of ~2–4 for stimulus strengths ~1–11, which leads to 10–20% increase in firing rates compared to 0 modulation strength. However, it is important to note that in any network model, stimulus strength and the strength of top-down modulations are only meaningful in the context of the parameters that govern spiking activity in the network (e.g., the dynamic range of the neurons, the slope of the activation functions, the degree of convergence of connections, the relative size of the each layers, etc.). As a result, we opted to parametrically sample a large range of stimulus strengths and attentional modulation levels to map out the full space of responses. Importantly, spanning the full range of values demonstrates that our general conclusions regarding random connections hold except in the most extreme cases (i.e., high stimulus/attentional strength levels where the system saturates and has little-to-no dynamic range).

In conclusion, our findings suggest that random connections provide a mechanism to relay highly selective top-down attentional modulations in a system where ‘control’ neurons exhibit mixed-selectivity. When the connections are sufficiently random, feedback signals only target relevant stimuli to enhance their sensory-evoked response pattern. In contrast, feedback signals related to other irrelevant features that are encoded by higher-order neurons largely cancel out and do not form representations that might be confusable with the attended stimulus. Finally, adding some structure to the connections, but only a modest amount, can reproduce empirically observed phenomena like the global spread of feature-based attention to unstimulated neurons tuned to the relevant feature. Together, these results emphasize the importance of balancing the random and structured connectivity when relaying targeted top-down feedback signals in a hierarchical network.

## Methods

### Spiking model

The model was implemented in Python using the Brian2 [152] simulation environment, and was based initially on the open-source code provided by Bouchacourt and Buschman (2019) [35]. The model consisted of two layers of Poisson spiking neurons, randomly and reciprocally connected to each other (see [35] and [153]). For all neurons, the synaptic activation *s* of each neuron *i* changes over time t:


$$\begin{array}{c} {\dot{s}}_{i}+\frac{s_{i}}{\tau }=\sum_{\alpha }\delta \left(t-t_{i}^{\alpha }\right) \end{array}$$ (1)

where ${\dot{s}}_{i}$ is the rate of change of $s_{i}(t)$, $\frac{s_{i}}{\tau }$ represents the exponential decay over time with synaptic time constant τ, $t_{i}^{\alpha }$ are the spike times of neuron *i*, and δ is Dirac delta function that represents the timing of the spikes for neuron *i*. Following previous studies and for simplicity the synaptic time constant τ was set to 10ms for all synapses [153,154]. Each neuron generates spikes based on the rate $g_{i}(t)$:


$$\begin{array}{c} g_{i}(t)=\;\sum_{j}W_{ij}s_{j}(t) \end{array}$$ (2)

where *Wij* is the strength of the connection from pre-synaptic neuron *j* to post-synaptic neuron *i* and $s_{j}(t)$ is the current activation state of pre-synaptic neuron *j*. Following reference [35], we used a baseline-shifted hyperbolic tangent as the nonlinear transfer function determining the relationship between a neuron’s activation state and spiking rate:


$$r_{i}=\;0.4(1+tanh(0.25g_{i}-3))$$ (3)

$$spikes(t+dt)\;\sim \;Poisson(r_{i}(t))$$ (4)

where $g_{i}$ is the total synaptic input for neuron *i* as determined by equation (2). This function is strictly positive and saturates at an upper bound, just like biological neurons. Note that we used a transfer function with a shallower slope (0.25) compared to Bouchacourt & Buschman (2019) (0.4) so that spiking rates in our model would exhibit a larger dynamic range to support fine-grained measurements of graded attentional modulations. While synaptic connections could be either excitatory or inhibitory, this model does not contain different cell-types and neurons do not have refractory periods following each spike. In addition, we did not explicitly inject noise as the inhomogeneous Poisson process that generates spikes naturally leads to variability in spike trains.

The input layer of the model contained 8 ring-like “sensory” sub-networks each composed of 512 neurons. Each sub-network contained neurons that encode information across a circular stimulus space such as orientation, motion, or color, and each sub-network can be interpreted as encoding these features in a distinct region of a retinotopic map (e.g., each sub-network encodes orientation information from a distinct location in the visual scene). Thus, every neuron in a sub-network had a preferred stimulus input, defined as an angle $\theta_{i}=2\pi i/N_{first}$ where $N_{first}$ is the number of neurons in each sub-network (512). This tuning arises due to short-range excitatory connections and long-range inhibitory connections between each neuron and its neighbors around the ring, where the synaptic weight between a pair of neurons *i*, *j* within a sub-network depends on $\theta =\theta_{i}-\theta_{j}$ through a difference of circular normal functions:


$$W_{ij}^{first}=w(\theta )=\lambda +Aexp(k_{1}(cos\theta -1))-Aexp(k_{2}(cos\theta -1))$$ (5)

where $k_{1}$= 1 determines the width of the excitation kernel, $k_{2}$ = 0.83 determines the width of the suppression kernel, A = 2 is the amplitude and λ = 0.28 is the baseline, and there was no self-excitation (w(0)=0). The parameters were adopted from Bouchacourt & Buschman (2019), with the exception that we used a higher value for $k_{2}$, which narrowed the suppression kernel to allow a single sub-network to maintain representations for two stimuli simultaneously so that we could simulate feature-based attention. Finally, the sub-networks were not directly connected to each other but neurons in the 8 sub-networks indirectly interacted via feed-forward and feedback connections with neurons in the second layer.

The second layer was composed of 1024 PFC-like ‘control’ neurons that were randomly connected to the neurons in the 8 sensory sub-networks. All first layer sub-networks converged onto the second layer through a weight matrix ($W^{FF}$) that determined the feedforward connectivity. Then, each neuron in the second layer network provided reciprocal feedback to the same subset of neurons in the first layer via feedback connectivity matrix ($W^{FB}$) that was the transpose of the feedforward connectivity matrix. Thus, connections between neurons in the first and second layers were bi-directional, with the probability of an excitatory connection set to 0.35. The strength of the excitatory feedforward and feedback connections were defined by the parameters α and β, respectively which were set to α = 2100 and β = 200 [35].

To balance the total excitatory drive across neurons, the feedforward and feedback weights were scaled by the number of total inter-network excitatory connections ($N_{exc}$) formed by each neuron. In addition, an equal inhibitory weight was applied to all inputs for each neuron ($-\alpha \;/\;(8*N_{first})$) so that each neuron receives an equal amount of excitatory and inhibitory drive (i.e., $\Sigma_{j}W_{ij}^{FF}=\Sigma_{j}W_{ij}^{FB}=0$). This was to mimic the effect of local inhibitory interneurons causing a broad inhibition in the network.

After this balancing process, excitatory feedforward connections from neurons in the first layer to a second layer neuron *i* had weight $W_{i,exc}=(\alpha /N_{{exc}_{i}})-(\alpha /(8*N_{first}))$ while inhibitory feedforward connections had weight $W_{i,inh}=\;-\alpha /(8*N_{first})$. When relaying feedback from the second to the first layer, neuron *j* in a sensory sub-network receive excitatory feedback connections with weight $W_{j,exc}=\;(\beta /N_{{exc}_{j}})-(\beta /N_{second})$ and inhibitory feedback connections with weight $W_{j,inh}=\;-\beta /N_{second}$.

### Stimulus design and input to sensory sub-networks

Sensory stimuli were provided as external synaptic drive ($s_{ext}$) to neurons in the first layer sub-networks, mimicking afferent inputs from the retina to visual cortex. The response of a given first layer neuron *i* at a given time *t* was thus a function of top-down feedback from *t*he second layer, the current state of other neurons in the same sensory sub-network, and external stimulus drive:


$$\begin{array}{c} r_{i}^{first}(t)=\Phi (\sum_{j\in second}W_{ij}^{FB}s_{j}(t)\;+\sum_{j\in first}W_{ij}^{first}s_{j}(t)+s_{i}^{ext}(t)) \end{array}$$ (6)

Stimulus inputs were presented for 300ms in the “sensory” task and for 1000ms in the “attention” task, indicated by the green and red shaded regions in Fig 2A, 2B, respectively. External stimulus inputs were generated using a circular normal distribution centered on an angle (μ) between 1° and 360°, with the input to neuron *i* given by:


$$s_{i}^{ext}=\left\{S^{ext}\frac{exp(\kappa \;cos(\theta_{i}\;-\;\mu ))}{2\pi I_{0}(\kappa )}\;if\;i\leq [3\sigma ],\;0\;if\;i\;>[3\sigma ]\right\}$$ (7)

where $\theta_{i}$ is the preferred stimulus input of neuron *i,* and κ determines the concentration, which was set to κ = 14 to approximate the shape of the Gaussian input in Bouchacourt & Buschman (2019). The standard deviation σ was determined based on the concentration parameter κ and our use of a circular normal input as opposed to a Gaussian input had no meaningful impact on the results. Inputs above and below three standard deviations were set to zero. $S^{ext}$ was the strength of external sensory input that was modulated based on the task between 0 and 19 (see next section for more detail). When multiple stimuli were presented to a single first layer sub-network in the attention task, they were always 180° apart.

### Applying top-down attention to the second layer

Top-down attentional modulation was provided as synaptic drive ($s_{att}$) to neurons in the second layer based on their selectivity for the attended feature. Thus, the response of a given second layer neuron *i* at a given time *t* was:


$$\begin{array}{c} r_{i}^{second}(t)=\Phi (\sum_{j\in first}W_{ij}^{FF}s_{j}(t)+S_{i}^{att}(t)) \end{array}$$ (8)

where $S^{att}$ was the strength of top-down attentional modulation that was modulated based on the task between 0 and 18. See S6 and S7 Figs for versions of the main Figs 2 and 3 with multiplicative, instead of additive, attentional modulations.

To identify the second layer neurons to target with top-down attentional modulation $S^{att}$, we used a *sensory* task where one of 16 possible stimulus features was presented in isolation to a single sub-network for 300ms, with 100 trials for each stimulus (Fig 2A). Then we rank-ordered the average firing rates of the second layer neurons during the stimulus presentation epoch and selected the top 20% that showed the highest firing rates to the stimulus that would be relevant in the subsequent attention task. Note that selecting different proportions of neurons to target with top-down attentional modulation only changed the strength of the effects, not the qualitative pattern of results (S4 and S5 Figs). Next, in the *attention* task, two stimuli were presented to a single sub-network for 1000 ms and attentional modulation was concurrently applied to the 20% of second layer neurons that exhibited a preferential response to the attended stimulus as defined using the method described above (Fig 2B). We varied the stimulus input strength between 0 and 19 in increments of 1, and top-down feature-based attentional modulation between 0 and 18 in increments of 2. Note that the range of stimulus strength and attentional modulations used is only meaningful in the context of the parameters that govern spiking activity in the network (e.g., the dynamic range of the neurons, the slope of their activation functions, the degree of convergence of connections, the relative size of the ‘sensory’ and ‘random’ layers, etc.) As a result, we tried to span the full range of stimulus strength and attentional modulation strength from 0 to driving the system into a nearly saturated state. We simulated 50 trials for each condition.

### Modulating randomness of the between-layer connections

To test the effect of randomness in mediating top-down modulations, we parametrically modulated the degree of structure in connections between neurons in the first and second layers. First, we determined the number of connections between each second layer neuron and neurons in each of the first-layer sub-networks using the same probability of forming an excitatory connection described above (0.35). Next, for every second layer neuron, a stimulus feature was randomly chosen as that neuron’s preferred stimulus μ. Then, we determined which neurons in the first layer would be connected to a given second layer neuron using a circular normal probability distribution function. This function spanned all possible features θ that were represented by neurons in each sub-network of the sensory layer and was centered on a selected preferred feature μ of each second layer neuron:


$$p(\theta )=\frac{exp(\kappa \;cos(\theta \;-\;\mu ))}{2\pi I_{0}(\kappa )}$$ (9)

where $I_{0}$ is the Bessel function of the first kind and the width of the probability distribution was determined by concentration parameter κ, which we modulated between 0 and 0.4 in increments of 0.1 (Fig 4). When κ = 0, the probability distribution was uniform and every neuron in each sub-network had an equal probability of being connected to a given second layer neuron. When κ > 0, the probability distribution was a bell-shaped curve and first layer neurons with similar preferred features had a higher chance of being connected to the same second layer neuron. Note that this process only impacted whether a certain between-layer connection existed and not the strength of the connection. As a result, when κ was higher, a second layer neuron was connected to sensory layer neurons that had similar tuning across sub-networks, causing second layer neurons to inherit lower-dimensional selectivity for stimulus features.

To test the effect of changes in κ, and thus changes in the randomness of connections in the network, we simulated feature-based attention as before using networks with varying κ levels. Critically, we compared feature-related signals for the attentional target from the stimulated and unstimulated sub-networks to test if there was an increase in the signal for the attended feature even in the sub-networks where no stimulus was presented.

### Evaluating the effects of stimulus strength and top-down attention

To quantify stimulus- and attention-related signals in each of the eight sensory sub-networks, the firing rates for each neuron were averaged across the last 250ms of each trial. We then separately averaged the firing rates of the two neurons centered on the attended and on the unattended stimuli to generate ‘contrast’ response functions (CRFs, but with stimulus strength standing in for actual stimulus contrast). To estimate the contrast gain of the CRFs, we fit a Naka-Rushton function [155,156]:


$$R(c)\;=\;G_{r}\frac{c^{n}}{c^{n}+G_{c}^{n}}+b_{c}$$ (10)

where c is normalized stimulus strength (0≤c≤1), $G_{r}$ is the multiplicative response gain, $G_{c}$ is the contrast gain that controls the horizontal shift of the CRFs, b is the response baseline offset, and n is the exponent that controls the speed at which the CRF rises and reaches asymptote. The fitting procedure was done with $G_{c}$ and n as free parameters and $G_{r}$ and b as fixed parameters: $G_{r}$ fixed to the difference between the highest and lowest firing rate and b fixed to the lowest firing rate for each CRF. There were two steps in the curve fitting procedure. First, we performed an initial grid search to find the best set of the two free parameters that yields the minimum root mean squared error. Then, to fine-tune the search, we used the SciPy ‘optimize’ function to minimize the root mean squared error between the data and the fit function with the best parameter set from the grid search as the initial seed for the search. The free parameters were restricted to the range of -10 and 10 for n, with the step size 0.1 for the grid search, and -1 and 1 for $G_{c}$, with the step size 0.01 for the grid search.

### Decoding analysis

To understand the nature of the feedback signals that were propagated to the first layer sub-networks in the feature-based attention simulations, we used a decoding method with a circular regression model to compare bottom-up stimulus-driven signals and signals modulated by top-down attentional feedback. We used BayesianRidge with default parameters in scikit-learn [157] with RegressorChain to fit the model to both sine and cosine of the stimulus angle. To ensure that the circular regression model that we used was able to learn the signals for different stimulus features and make accurate predictions on which stimulus was presented, we trained and tested the model on the *sensory* task data for the stimulated sub-network. The training and test data were comprised of the last 250ms of spiking activity, and were split so that one trial for each presented stimulus was assigned to the test data and the rest of the trials were assigned to the training data. Since there were 100 trials for each stimulus and 16 stimuli total, this led to 16 trials in test data and 1584 trials in training data. Then, the circular regression model with empirical Bayesian ridge regression was fit to the training data to predict which stimulus was presented in the test data. We took the absolute difference between the predicted and the actual stimulus value and averaged across all trials to obtain the mean absolute error (MAE). We repeated this procedure so that all trials had a chance of being included in the test data and averaged the resulting MAE values over all cross-validation folds.

Next, we sought to determine if the top-down attentional modulations resulted in stimulus-specific representational changes related to the attended stimulus. We used a cross-generalization approach in which the regression model was trained on all of the data from the *sensory* task and tested on data from the *attention* task data to predict which stimulus feature was attended in the stimulated sub-network (1600 sensory task trials in training data and 100 attention task trials in test data). This analysis was based on the logic that if the attentional feedback signals lead to changes in signals that are stimulus-specific, resembling stronger stimulus input, the regression model trained on the presented stimulus in the *sensory* task will successfully predict the attended stimulus in the *attention* task. Average MAE was calculated by taking the average absolute difference between the predicted stimulus value and the attended stimulus value on each trial and averaging across all trials. Since the stimulus strength was fixed at 10 for the *sensory task* but varied from 0 to 19 in the *attention* task, we also calculated MAE for each stimulus strength level separately (*Control analysis for different stimulus strength* in S1 Text).

In addition to this sensory-to-attention comparison, we sought to understand the nature of signals propagated to the unstimulated sub-networks via top-down modulations relayed by second layer neurons with high-dimensional selectivity. First, we tested to see if the feedback signals sent to unstimulated sub-networks were consistent for a given attended feature, regardless of whether these signals resembled stimulus-driven responses. Thus, we trained a linear support vector machine (SVM) [158] to classify the attended stimulus value on each trial in the *attention* task, using SVC with default hyperparameters in scikit-learn [157]. We then performed cross-validation by leaving out one trial for each of the two stimuli that could be attended. Data from the remaining trials were then used to train the classifier, and this process was repeated until every trial had been assigned to the training and test sets. Since there were 50 trials for each stimulus and 2 stimuli total, this led to 2 trials in test data and 98 trials in training data for each iteration. Classifier accuracy was then averaged across all cross-validation folds. This procedure was performed separately for each of the 8 first layer sub-networks and each stimulus strength level.

Second, we wanted to test if attentional feedback signals led to patterns of activity that were systematically related *across* the unstimulated sub-networks. We trained a SVM classifier on activity patterns in each unstimulated sub-network to classify which stimulus was attended in the stimulated sub-network and then tested this classifier on data from a different unstimulated sub-network. This cross-generalization between activity patterns in unstimulated networks revealed how well attention-related activity patterns learned by the classifier transferred between two unstimulated sub-networks. We then looped over the seven unstimulated sub-networks, using data from each sub-network as a training dataset and testing on each of the remaining six unstimulated sub-networks before averaging decoding accuracy across all iterations (100 trials in training, 100 trials in testing for each iteration).

The same cross-generalization procedure was also applied to assess representational changes with different levels of randomness in the between-layer connections (Fig 5, bottom row).

### Network initializations

To ensure that the effects we observed in the simulations were not due to a specific assignment of connectivity weight matrices, we repeated all simulations across ten iterations using different random seeds to initialize the network. Importantly, this gave rise to a different connectivity pattern and weights between the first and the second layer in each initialization, and all plots show averaged data across these ten iterations with the shaded region as the standard error of mean.

## Supporting information

S1 Text Supporting Information including control analyses and results and S1–S7 Figs. (DOCX)

S1 Fig Average MAE from Fig 3B separated by stimulus strength (SS) level, as indicated above each plot. Solid black lines represent MAE in the stimulated sub-network and dotted gray lines represent MAE in the unstimulated sub-network. Shaded areas represent standard error of mean across 10 different network initializations.
(TIF)

S2 Fig Average decoding accuracy from Fig 3C separated by stimulus strength (SS) level, as indicated above each plot. Solid black lines represent decoding accuracies in the stimulated sub-network and dotted gray lines represent decoding accuracies in the unstimulated sub-network. Shaded areas represent standard error of the mean across 10 different network initializations.
(TIF)

S3 Fig Comparison of raster plots for the *sensory* task with stimulus strength at 10 and *attention* task with stimulus strength at 12 and feature-based attention modulation strength at 6. In this example, the presented stimulus in the *sensory* task and the attended stimulus in the *attention* task was 90°. However, note that the pattern associated with the unattended stimulus at 270° more closely resembles the pattern associated with the sensory evoked response (if it was centered at 270°) than an attended response because the response to attended stimuli is more dispersed.
(TIF)

S4 Fig Feature-based attention simulations with different proportions of second layer neurons receiving top-down modulation, as indicated above each plot. **A**. Example trials from the attention task for each proportion. **B**. Response functions when the stimulus was attended (solid lines) and unattended (dotted lines). Each line color represents a different level of feature-based attention modulation strength. **C**. Estimated contrast gain parameters from attended (solid black line) and unattended (dotted gray line) conditions. These plots are from a single network initialization and therefore do not contain any error areas.
(TIF)

S5 Fig SVM Decoding results from simulations with different proportions of second layer neurons receiving top-down modulation, as indicated above each column, and for different level of stimulus strength, as indicated to the left of each row. Solid black lines represent decoding accuracies in the stimulated sub-network and dotted gray lines represent decoding accuracies in the unstimulated sub-network. These plots are from a single network initialization and therefore do not contain any error areas.
(TIF)

S6 Fig Feature-based attention simulations with a multiplicative attention modulation. **A**. An example trial from the attention task. All parameters were same as the data shown in Fig 2B, except that the feature-based attention modulation was multiplicative instead of additive. Two stimulus inputs, at 90° and 270°, were presented to sub-network 1 and at the same time (stimulus strength: 6), feature-based attention modulation was applied to a subset of second layer neurons that have the highest selectivity to the 90° stimulus in sub-network 1 (lower cluster of spikes; modulation strength: 8) for 1000ms. B, C. Response functions when the stimulus was attended (**B**) and unattended (**C**). Each line represents a different level of feature-based attention modulation strength. Note that 1 is the lowest modulation value used as a baseline here, since the modulation is applied multiplicatively and not additively. **D**. Estimated contrast gain parameters from attended (solid black line) and unattended (dotted gray line) conditions. These plots are from a single network initialization and therefore do not contain any error areas.
(TIF)

S7 Fig Decoding results from feature-based attention simulations with multiplicative attention modulation. **A.** Histograms of the predicted identity of the attended stimulus for every trial in the *attention* task for the stimulated sub-network based on a circular ridge regression model trained on the *sensory* task, for a single network initialization. Each histogram represents predictions from different levels of feature-based attention modulation strength, as marked above each plot. **B.** Average MAE between the predicted and actual stimulus input for stimulated (solid black line) and unstimulated (dotted gray line) sub-networks based on regression model predictions. **C.** Decoding accuracy of support vector machines (SVMs) trained and tested on the *attention* task for Stimulated (solid black line) and Unstimulated (dotted gray line) sub-networks. **D.** Decoding accuracy of SVMs based on the *attention* task, trained on one unstimulated sub-network and tested on another unstimulated sub-network. These plots are from a single network initialization and therefore do not contain any error areas.
(TIF)
