# Supplementary material for: Near-random connections support top-down feature-based attentional modulations in early sensory cortex
Source: PLoS Comput Biol. 2025 Aug 12;21(8):e1013396. doi: 10.1371/journal.pcbi.1013396 (PMC12364372; doi:10.1371/journal.pcbi.1013396)
Supplement: S1 Text — (DOCX) [file pcbi.1013396.s001.docx]

**Random connections support precise top-down modulations of early sensory cortex: Supporting Information, S1 Text**

**Control analysis for different stimulus strength**

In the results presented in Figure 3, stimulus strength was fixed at 10 for the *sensory* task but varied from 0 to 18 in the *attention* task. Thus, we ran the decoding analyses separately for each stimulus strength level for the *attention* task (Supplementary Figure 1,2). For stimulus strengths 0 to 10, MAE increased as a function of feature-based attentional modulation and with higher stimulus strength (Supplementary Figure 1). However, when the stimulus strength was higher than 10, we observed that prediction accuracy decreased with higher feature-based attentional modulation, with MAE eventually rising above the chance level of 90°. This lower-than-chance prediction accuracy indicates that the regression model was systematically guessing the value of the unattended stimulus because the response pattern associated with a strong stimulus and strong attentional modulation diverged substantially from the sensory-evoked response that was used to train the regression model (as shown in the raster plots in Supplementary Figure 3). This divergence from the sensory-evoked response at high stimulus strengths thus led the model to predict that the unattended stimulus was more similar to the sensory signals that were used to train the model.

With the SVM decoding results, we find that decoding accuracy increases as a function of top-down attentional modulation strength across all levels of stimulus strength. However, at higher levels of stimulus strength, the decoding accuracy for stimulus sub-networks is lower than unstimulated sub-networks for lower attentional modulation strengths (SS = 13-19, attentional modulation < 10). This is likely caused by strong bottom-up input of the unattended stimulus disrupting the stability of feedback signals in the stimulated sub-network. Since unstimulated sub-networks were not provided any stimulus input, the pure feedback signals could be read out more consistently compared to the stimulated sub-network. When top-down attentional modulation is strong enough to overcome the competing stimulus input (> 10), we see that the decoding accuracies are similar between the stimulated and unstimulated sub-networks.

**Control analysis for different proportion of neurons that receive top-down modulation**

In the main simulations, we identified the top 20% of the second layer neurons that showed the highest firing rates to the stimulus as targets for top-down feature-based attentional modulation. We also varied the proportion of neurons selected from 10% to 50%, and found that the activity in the second layer increased naturally with as the proportion of neurons that received modulation increased (Supplementary Figure 4A). Importantly, across all levels we stimulation, we observed a similar pattern of results as reported in the main Figures 2 and 3, with the effect of attentional modulations being more pronounced when a higher proportion of second layer neurons were stimulated. For example, when the stimulated proportion was higher, the contrast gain parameter decreased faster as a function of feature attention modulation (Supplementary Figure 4C) and decoding accuracy increased faster as a function of feature attention modulation in the stimulated sub-network when stimulus strength was low (Supplementary Figure 5). In addition, we found that for the unstimulated sub-networks, when the stimulus strength was high enough (SS = 15), SVM decoding was slightly higher when a lower proportion of the second layer neurons received attentional modulation compared to when the proportion was higher. In contrast, when the stimulus strength was lower, driving fewer second layer neurons may not have been sufficient to induce stable feedback in the unstimulated sub-networks, which led to lower decoding accuracy. Thus, there seems to be a trade-off: Stimulating fewer second layer neurons results in better decoding of the attended feature based on activity in the unstimulated sub-networks, but only when the signal-to-noise ratio is sufficiently high.

**Control analysis for multiplicative top-down modulation**

In the main simulations, we applied additive top-down attentional modulation as described in Equation 8. We also ran simulations using a multiplicative gain following this equation:

$r_{i}^{second}(t)=\Phi\left( \sum_{j\in first} W_{ij}^{FF}s_{j}(t){*S}_{i}^{att}(t) \right)$ (1)

while varying the strength of top-down attentional modulation strength between 1 and 18. Note that since the top-down modulation is multiplied by the feedforward synaptic input, its baseline value is set to 1 instead of 0 as in the case of an additive modulation. As shown in Supplementary Figures 6 and 7, the effect of multiplicative top-down modulation showed a similar yet more pronounced effect of attentional modulation strength when compared to the pattern of results for the additive top-down modulations reported in the main text. However, our general conclusions do not change.
