# Supplementary figures and images for: Near-random connections support top-down feature-based attentional modulations in early sensory cortex
Source: PLoS Comput Biol. 2025 Aug 12;21(8):e1013396. doi: 10.1371/journal.pcbi.1013396 (PMC12364372; doi:10.1371/journal.pcbi.1013396)

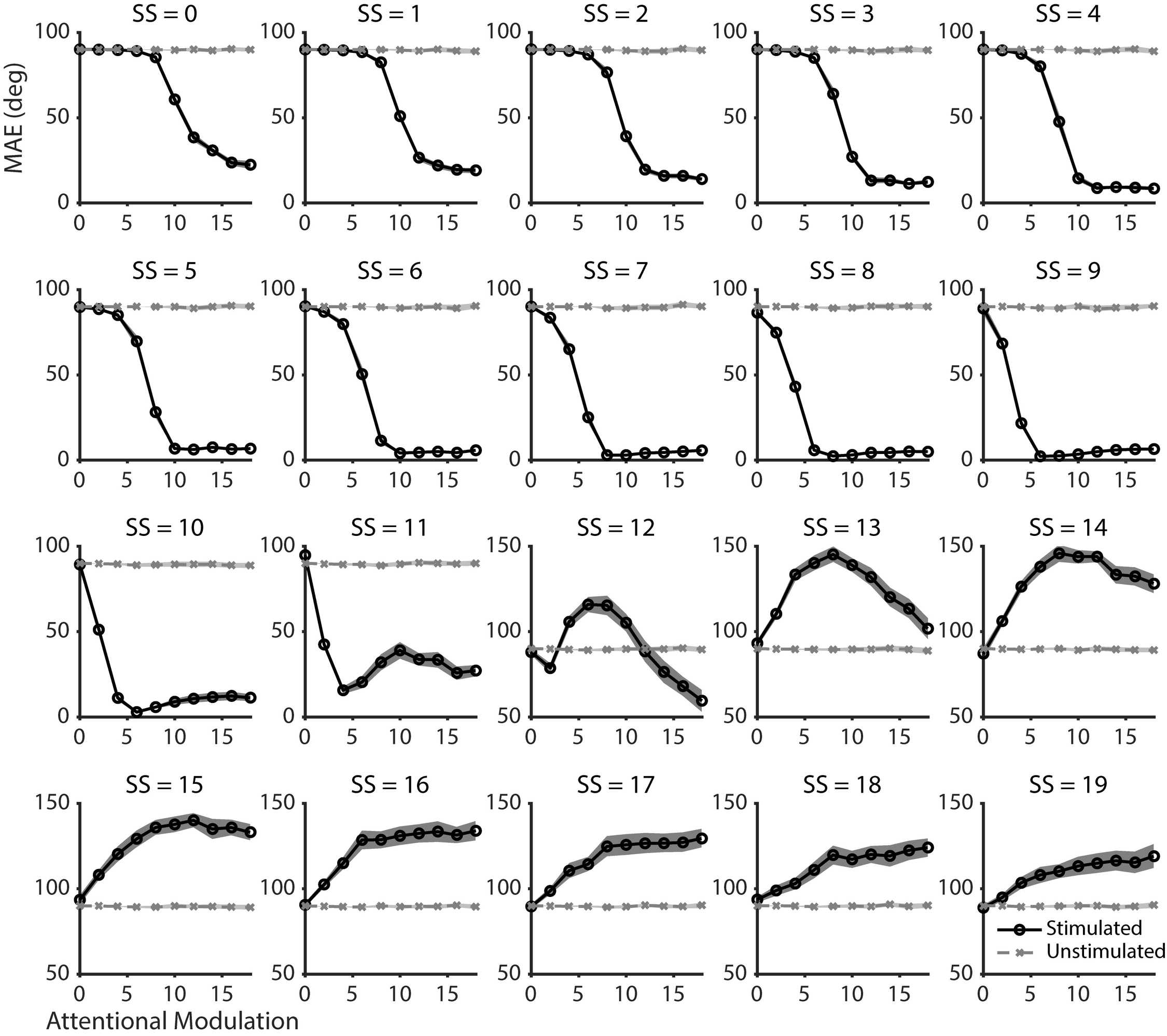

Supplement: S1 Fig — Solid black lines represent MAE in the stimulated sub-network and dotted gray lines represent MAE in the unstimulated sub-network. Shaded areas represent standard error of mean across 10 different network initializations. (TIF) [file pcbi.1013396.s002.tif]

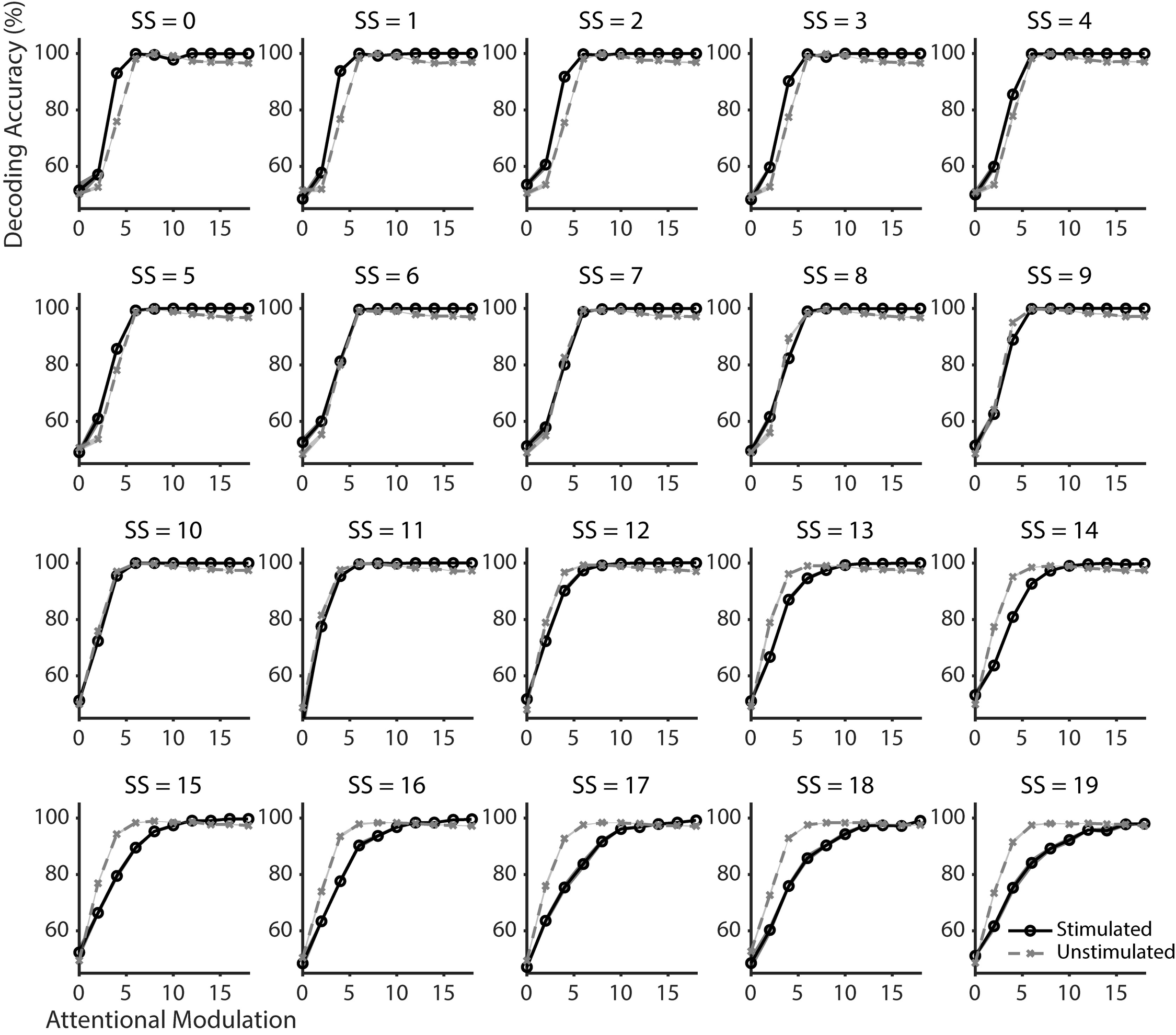

Supplement: S2 Fig — Solid black lines represent decoding accuracies in the stimulated sub-network and dotted gray lines represent decoding accuracies in the unstimulated sub-network. Shaded areas represent standard error of the mean across 10 different network initializations. (TIF) [file pcbi.1013396.s003.tif]

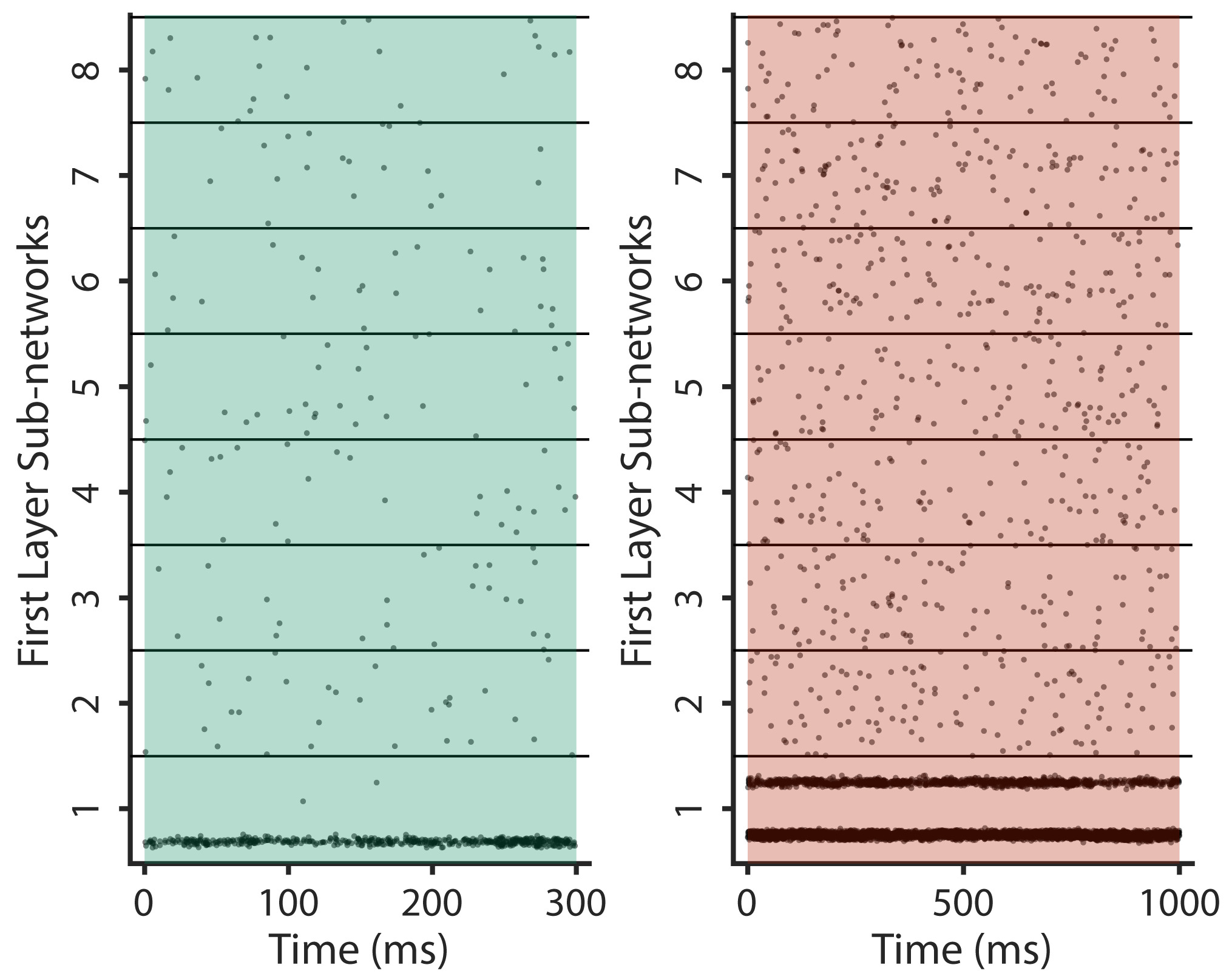

Supplement: S3 Fig — In this example, the presented stimulus in the sensory task and the attended stimulus in the attention task was 90°. However, note that the pattern associated with the unattended stimulus at 270° more closely resembles the pattern associated with the sensory evoked response (if it was centered at 270°) than an attended response because the response to attended stimuli is more dispersed. (TIF) [file pcbi.1013396.s004.tif]

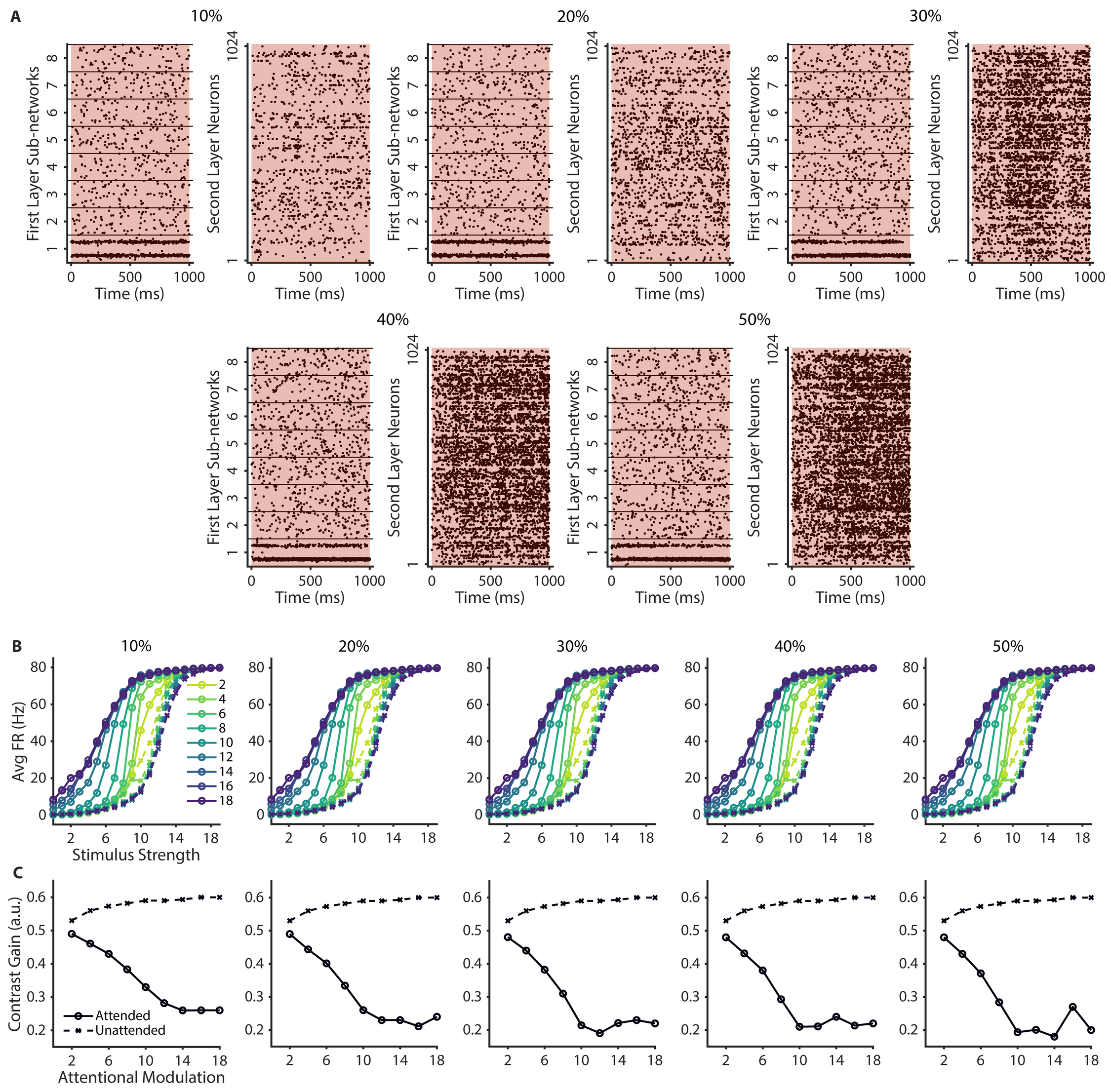

Supplement: S4 Fig — A. Example trials from the attention task for each proportion. B. Response functions when the stimulus was attended (solid lines) and unattended (dotted lines). Each line color represents a different level of feature-based attention modulation strength. C. Estimated contrast gain parameters from attended (solid black line) and unattended (dotted gray line) conditions. These plots are from a single network initialization and therefore do not contain any error areas. (TIF) [file pcbi.1013396.s005.tif]

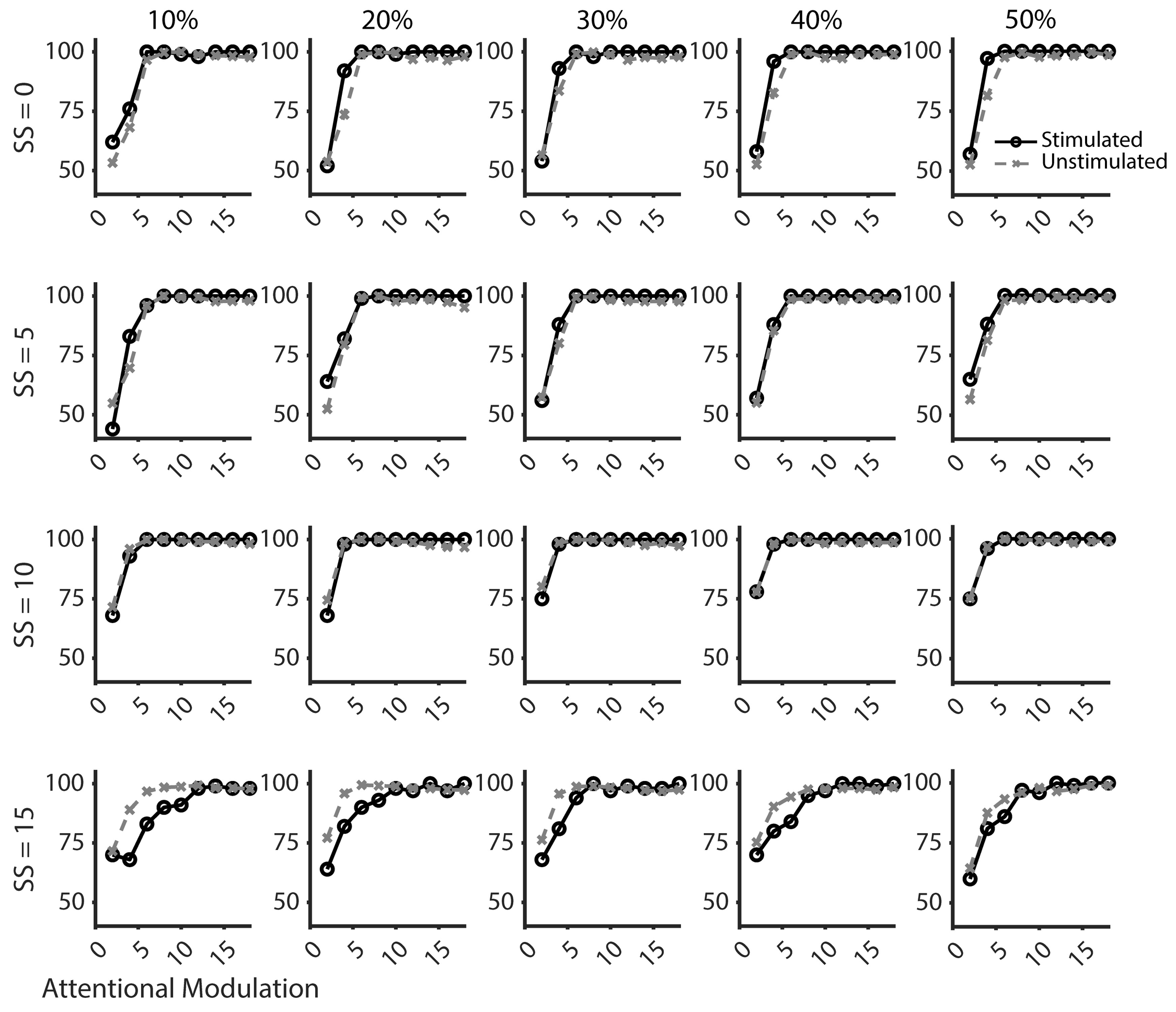

Supplement: S5 Fig — Solid black lines represent decoding accuracies in the stimulated sub-network and dotted gray lines represent decoding accuracies in the unstimulated sub-network. These plots are from a single network initialization and therefore do not contain any error areas. (TIF) [file pcbi.1013396.s006.tif]

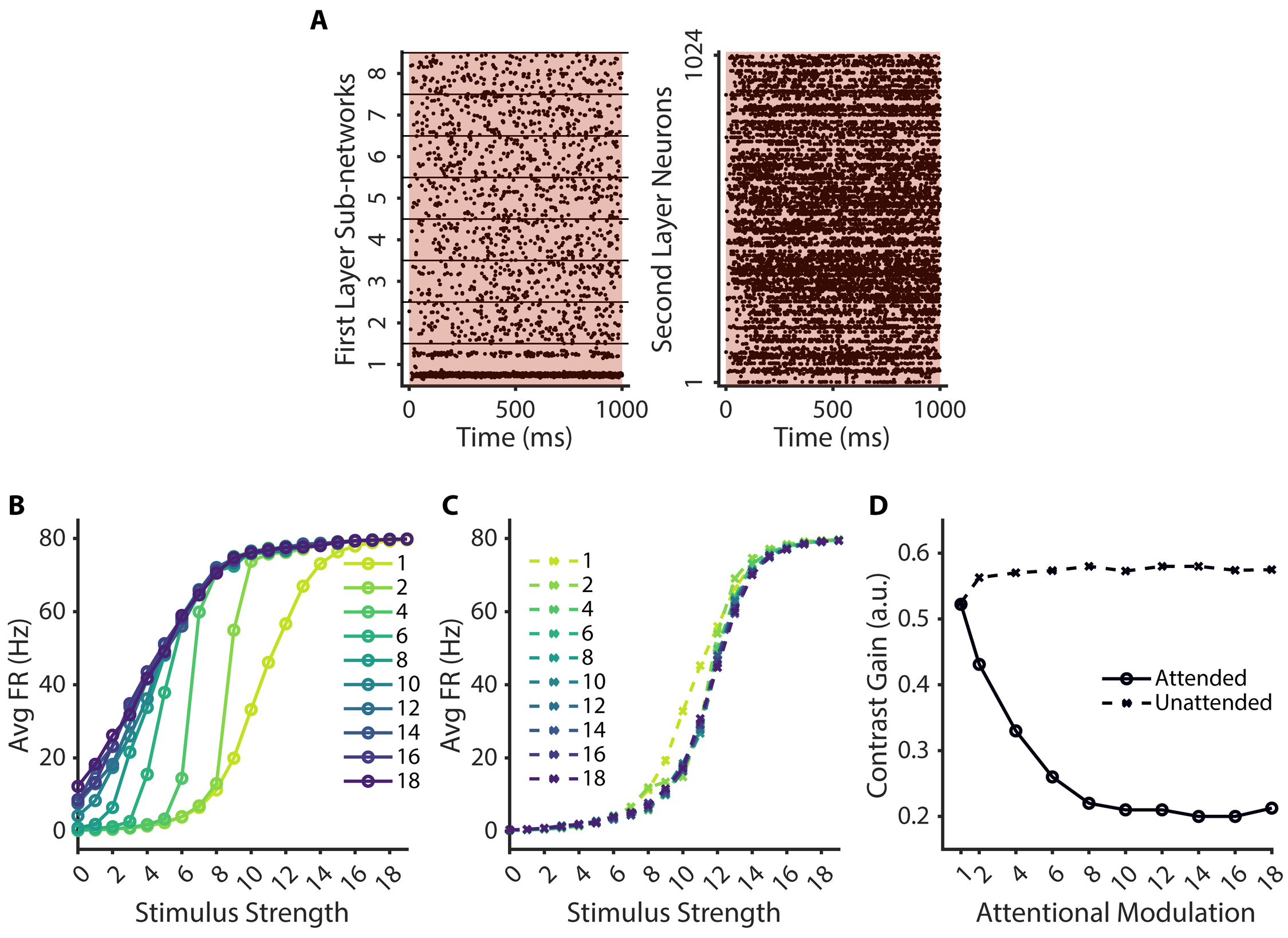

Supplement: S6 Fig — A. An example trial from the attention task. All parameters were same as the data shown in Fig 2B, except that the feature-based attention modulation was multiplicative instead of additive. Two stimulus inputs, at 90° and 270°, were presented to sub-network 1 and at the same time (stimulus strength: 6), feature-based attention modulation was applied to a subset of second layer neurons that have the highest selectivity to the 90° stimulus in sub-network 1 (lower cluster of spikes; modulation strength: 8) for 1000ms. B, C. Response functions when the stimulus was attended (B) and unattended (C). Each line represents a different level of feature-based attention modulation strength. Note that 1 is the lowest modulation value used as a baseline here, since the modulation is applied multiplicatively and not additively. D. Estimated contrast gain parameters from attended (solid black line) and unattended (dotted gray line) conditions. These plots are from a single network initialization and therefore do not contain any error areas. (TIF) [file pcbi.1013396.s007.tif]

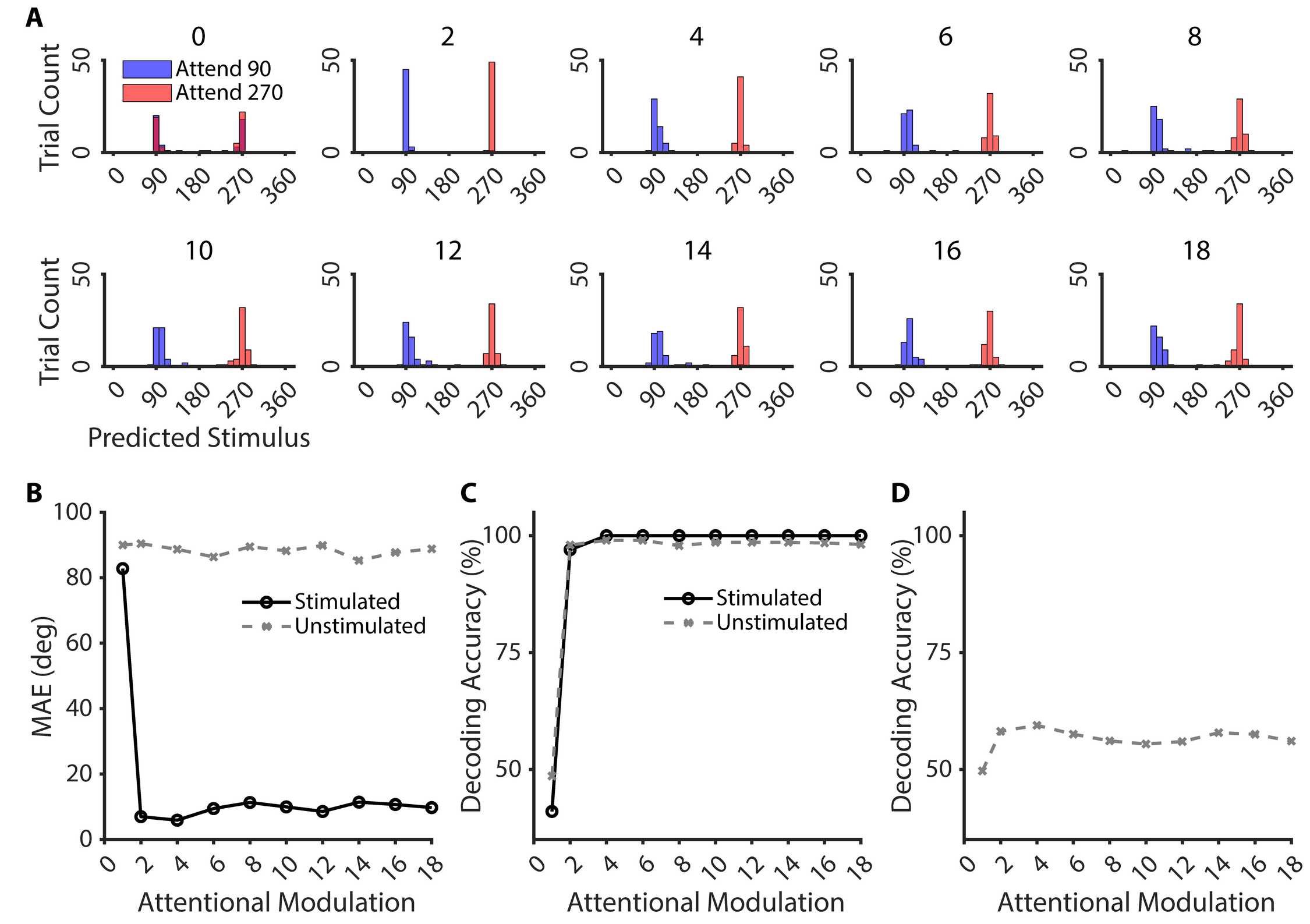

Supplement: S7 Fig — A. Histograms of the predicted identity of the attended stimulus for every trial in the attention task for the stimulated sub-network based on a circular ridge regression model trained on the sensory task, for a single network initialization. Each histogram represents predictions from different levels of feature-based attention modulation strength, as marked above each plot. B. Average MAE between the predicted and actual stimulus input for stimulated (solid black line) and unstimulated (dotted gray line) sub-networks based on regression model predictions. C. Decoding accuracy of support vector machines (SVMs) trained and tested on the attention task for Stimulated (solid black line) and Unstimulated (dotted gray line) sub-networks. D. Decoding accuracy of SVMs based on the attention task, trained on one unstimulated sub-network and tested on another unstimulated sub-network. These plots are from a single network initialization and therefore do not contain any error areas. (TIF) [file pcbi.1013396.s008.tif]
